# Leukocyte recruitment in flavivirus-induced encephalitis

**DOI:** 10.3389/fimmu.2025.1650903

**Published:** 2025-08-22

**Authors:** Emily Slowikowski, Céleste Willems, Pedro Elias Marques

**Affiliations:** Laboratory of Molecular Immunology, Department of Microbiology, Immunology and Transplantation, Rega Institute for Medical Research, Katholieke Universiteit Leuven (KU Leuven), Leuven, Belgium

**Keywords:** flavivirus, leukocyte, encephalitis, chemoattractant, chemokine, adhesion molecule

## Abstract

Flaviviruses are capable of causing a myriad of diseases in humans, including viral encephalitis. This condition involves complex interactions between the virus, resident cells of the central nervous system and leukocytes recruited to the brain. We reviewed the mechanisms underlying leukocyte recruitment during flavivirus-induced encephalitis with a focus on the role of various chemoattractants and adhesion molecules. We discuss how these molecules orchestrate the migration of peripheral leukocytes into the brain parenchyma and how neurotropic flaviviruses induce this process. Moreover, we discuss evidence of leukocytes both controlling viral propagation and contributing to neuropathology, which poses a challenge for therapy development. This review summarizes our current understanding of the mechanisms behind leukocyte recruitment during encephalitis, addresses the gaps that remain in the field, and presents opportunities for therapeutic targeting unveiled by recent research on flaviviral encephalitis.

## 1 Introduction

Flaviviruses are a group of arthropod-borne RNA viruses estimated to infect up to 400 million people annually. Even though most flavivirus infections occur asymptomatically or cause mild febrile disease, these viruses can cause a wide spectrum of severe visceral, neurotropic and congenital diseases (1). Neurotropic flaviviruses have developed the capacity to overcome the protective barriers of the central nervous system (CNS), allowing them to cause severe inflammation of the meninges and/or the brain parenchyma, referred to respectively as meningitis and encephalitis, conditions that lead to neurodegeneration, edema and intracranial hypertension. Morbidity and mortality rates associated with CNS infections are high, especially in young children, the elderly and immunocompromised individuals. Neurological sequelae comprise headaches, movement disorders, altered consciousness, seizures and cognitive impairment, which can last up to long after the acute infection (2, 3). Clinically important neurotropic flaviviruses are Japanese encephalitis virus (JEV), West Nile virus (WNV) and tick-borne encephalitis virus (TBEV). Infections caused by flaviviruses such as Usutu virus (USUV), St. Louis encephalitis virus (SLEV) and Murray Valley encephalitis virus (MVEV) are rare. However, neglected flaviviruses pose a significant public health threat due to underestimation, lack of vaccines or antivirals, and their emerging potential for outbreaks (1). Other flaviviruses that are primarily associated with visceral or congenital diseases, such as dengue virus (DENV) and Zika virus (ZIKV), also possess the capacity to induce neurological disease (4–6).

Upon infection of the brain, resident cells such as neurons, microglia and astrocytes initiate an immune response by secretion of proinflammatory cytokines and chemokines. Consequently, peripheral leukocytes including monocytes and lymphocytes are recruited to the CNS, where they support viral clearance (7, 8), however, it has become clear that immune-mediated tissue damage and neuronal loss play a significant role in encephalitis. Moreover, neurotropic flaviviruses can infect and exploit leukocytes for neuroinvasion via a ‘trojan horse’ mechanism (9). The effector functions and consequences of cellular infiltration have been reviewed extensively elsewhere, as were the entry mechanisms of flaviviruses into the brain (10–14). This review focuses on the molecular mechanisms by which peripheral leukocytes migrate to the brain upon flavivirus infections. We discuss the leukocyte subsets that are recruited and the involvement of multiple chemoattractants and cellular adhesion molecules (CAMs).

## 2 Leukocyte entry sites of the brain

**Figure 1:**
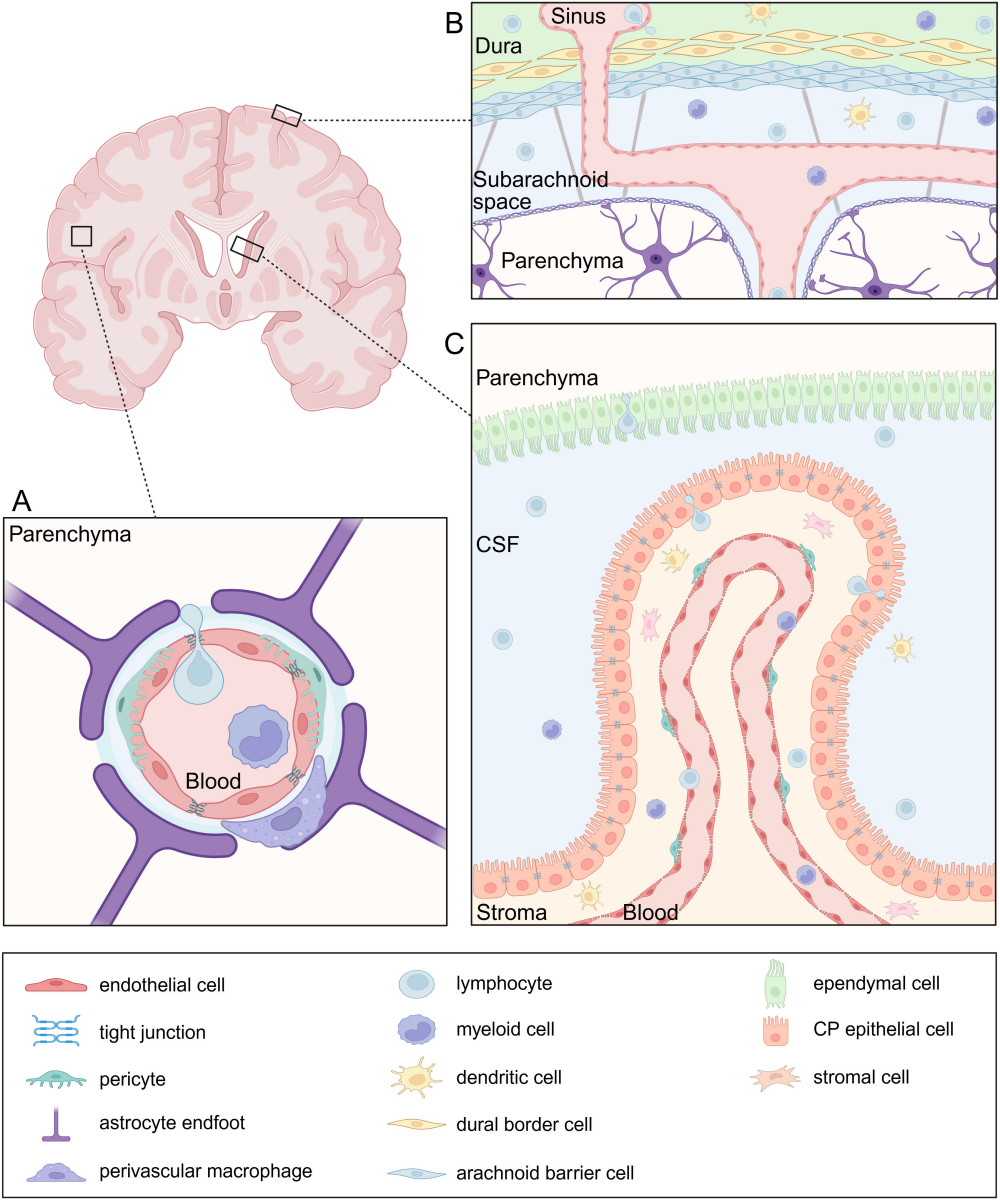
Neuroanatomy of leukocyte entry sites. **(A)** The blood-brain barrier (BBB) is composed of specialized endothelial cells connected by tight junctions (TJs), strictly regulating paracellular transport. Pericytes are embedded in the endothelial basement membrane which is distinct in composition from the parenchymal basement membrane. The latter forms the glia limitans together with astrocyte endfeet and serves as an additional barrier. At the level of the post-capillary venules, the basement membranes are separated by a perivascular space which holds antigen-presenting cells (APC) such as perivascular macrophages. **(B)** The meninges consist of three layers: the dura mater, arachnoid mater, and pia mater. Peripheral leukocytes infiltrate the dura through sinuses, which lack TJs. The arachnoid mater separates the dura from the cerebrospinal fluid (CSF)-filled subarachnoid space, which contains various immune cell populations. The pia mater together with the glia limitans form the last barrier to the parenchyma. **(C)** The blood-cerebrospinal fluid barrier (BCSFB) is located in the choroid plexus and is composed of an epithelial cell layer sealed by TJs and strictly regulating the entry of immune cells from the stroma into the CSF. APCs and T cells are abundant in the stroma which they can easily enter from the blood through a fenestrated endothelium.

Specialized barriers separate physically the CNS from the periphery, posing a challenge for immune cells to reach the tissue. The blood-brain barrier (BBB) is undeniably the most well-known and studied barrier, consisting of a specialized endothelial layer that strictly controls the entry of solutes and immune cells from the blood into the brain parenchyma (**Figure 1A**). Nevertheless, leukocytes enter the brain via both paracellular and transcellular mechanisms. The paracellular route involves leukocyte migration between endothelial cells through junctional complexes. This process is facilitated by the interaction between leukocytes and endothelial cells through adhesion molecules such as intracellular adhesion molecule-1 (ICAM-1) and ICAM-2, as well as integrins like αLβ2 and αMβ2, which drive leukocyte crawling and their eventual passage through junctions (15). Tight junctions (TJ) between the endothelial cells strictly control paracellular transport and consist of transmembrane proteins such as occludin, claudins and junctional adhesion molecules (JAMs). This TJ backbone recruits several cytoplasmic scaffolding proteins, such as zona occludens, which contribute to TJ integrity. More specifically, claudin-5, the most abundant TJ protein in BBB endothelial cells, has been proposed to facilitate T-cell diapedeses across the BBB (16, 17). The regulation of TJ and BBB is profoundly dependent on the neurovascular unit, comprising astrocytes, perivascular microglia, pericytes and neurons (18, 19). Additionally, the endothelial and parenchymal basement membranes form another physical barrier to infiltrating leukocytes and are produced by endothelial cells and astrocytes, respectively.

The transcellular route involves leukocytes migrating directly through endothelial cells rather than between them. This route is characterized by the formation of vesiculotubular structures, intraendothelial structures or dynamic pores within endothelial cells (20). During transcellular diapedesis, endothelial cells form protrusions that surround the leukocyte, creating cup-like structures that facilitate the formation of temporary channels or pores, allowing leukocyte migration across the BBB while maintaining the TJ integrity (21). The molecular mechanisms directing paracellular and transcellular leukocyte diapedeses across the BBB are not yet understood. Research suggests that different leukocyte types may favor one route over the other based on their interactions with CAMs and the specific inflammatory context.

While the meninges are traditionally viewed as a protective barrier for the brain parenchyma, growing evidence highlights the role of meningeal immunity as a key contributor to CNS homeostasis and inflammation (**Figure 1B**). The meninges are composed of three layers, the outermost dura mater, the arachnoid mater and the innermost pia mater. Leukocytes access the dura from the circulation by extravasation through the dural sinuses which lack TJs. In addition, the skull bone marrow serves as a local source of leukocytes that migrate directly into the adjacent dura independently from the circulation. The dural immune cell population is heterogeneous and mainly consist of macrophages and lymphoid cells. The arachnoid mater separates the dura from the cerebrospinal fluid (CSF)-filled subarachnoid space, which houses a variety of immune cells. However, the blood vessels within the subarachnoid space restrict leukocyte extravasation (22, 23). Recently, arachnoid cuff exit points, which are gaps surrounding the bridging veins delineated by arachnoid barrier cells, have been identified as an additional route for leukocyte entry into the CNS. These exit points facilitate the direct efflux of CSF from the subarachnoid space to the dura and the entry of immune cells in reverse direction (24).

Another interface between blood and the CNS is the blood-cerebrospinal fluid barrier (BCSFB) (**Figure 1C**). CSF is produced by choroid plexus epithelial cells (CPE) within the four brain ventricles. The choroid plexus (CP) is lined by a fenestrated endothelium through which fluids, solutes and leukocytes easily pass in both homeostatic and encephalitic conditions. Moreover, the ependymal cells that separate the ventricles from the brain parenchyma contain solely loose junctions forming a leaky CSF-parenchyma interface. However, the CP epithelium itself serves as barrier and contains TJs that limit leukocyte passage (25–28). Importantly, the stroma contains a variety of innate and adaptive immune cells, including memory CD4^+^ T cells, which enter the CSF through the epithelium in physiological conditions and exert an important function of CNS immune surveillance (28, 29).

## 3 Chemokines and chemokine receptors

A hallmark of CNS infections is the infiltration of leukocytes into the brain parenchyma and the CSF, also referred to as pleocytosis. While viral CNS infections are characterized by lymphocytic predominance, several studies on WNV and TBEV infections point to neutrophils predominating in the earliest days following the onset of symptoms (30–33).

A key prerequisite for leukocyte recruitment is the formation of a chemokine gradient. Chemokines are small, secreted proteins that direct leukocyte migration by binding to glycosaminoglycans (GAGs) on endothelial cells and activating G protein-coupled receptors (GPCRs) on leukocytes. Based on the position of conserved amino-terminal cysteine residues, chemokines are structurally classified into four subgroups (CC, CXC, CX3C and XC) (34).

**Figure 2:**
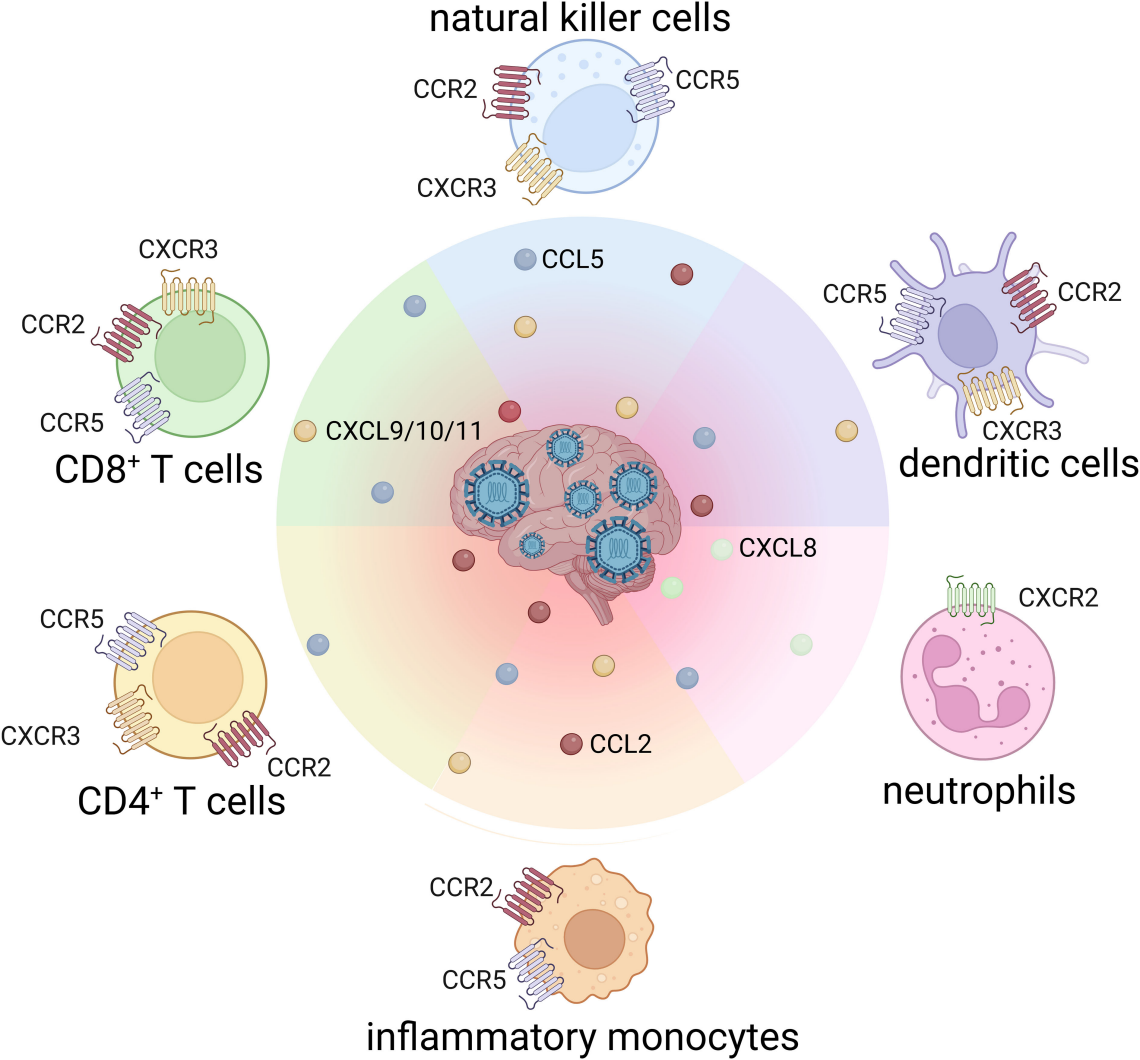
Chemokine receptor profiles on leukocytes driving CNS infiltration in flaviviral encephalitis.

In the healthy CNS, several chemokines are constitutively expressed to support immune surveillance. However, during viral infection, their expression is strongly upregulated in neurons, astrocytes and microglia (**Table 1**). Elevated chemokine levels detected in the CSF of patients with viral encephalitis may serve as diagnostic or prognostic biomarkers (35–37). Likewise, the expression of chemokine receptors on infiltrating leukocytes is increased in response to neurotropic infections (**Figure 2**).

**Table 1:** Expression of chemokine receptors and their cognate ligands in the CNS upon neurotropic flavivirus infection *in vivo*.

Receptor	Expression	Virus	Measurement	Model	Reference
**CCR2**	**↑**	JEV	mRNA level (8dpi)	BALB/c mice, 3–4 w old, male and female, were iv inoculated (tail-vein injection) with 3 x 10^5^ PFU of JEV (GP78 strain)	(46)
	**↑**	JEV	mRNA level	C57BL/6 J mice, 5–6 w old, male and female, were iv inoculated with 1.7–5.0 x 10^6^ PFU of JEV (P3 strain)	(65)
	**↑**	WNV	mRNA level (7 and 12 dpi)	C57BL6/J mice, 8–12 w old, female, were sc inoculated with 10^4^ FFU WNV (NY99–35262 strain)	(43)
	**↑**	WNV	Protein level (7 and 25 dpi)	C57BL6/J (*Tmem119^GFP/+^Ccr2^RFP/+^*) mice, 8–12 w old, male and female, were ic inoculated with 10^4^ PFU WNV (WNV-NS5-E218A strain)	(53)
CCR2 LIGANDS					
CCL2	**↑**	JEV	mRNA level	C57BL/6 J mice, 5–6 w old, male and female, were iv inoculated with 1.7–5.0 x 10^6^ PFU of JEV (P3 strain)	(65)
	**↑**	JEV	Protein level (9 dpi)	BALB/c mice, 4–6 w old, male and female, were iv inoculated (tail-vein injection) with 3 x 10^5^ PFU of JE virus (GP78 strain)	(44)
	**↑**	JEV	Protein level (4 dpi)	Wistar strain rats, 12 d old, male and female, were ic inoculated with 3 x 10^6^ PFU of JE virus (GP78 strain)	(45)
	**↑**	USUV	Protein level (6 dpi)	C57BL6/J mice, 8–12 w old, male and female, were ic inoculated with 10^4^ PFU USUV (MK 230892 strain)	(52)
	**↑**	WNV	mRNA level (7 and 12 dpi)	C57BL6/J mice, 8–12 w old, female, were sc inoculated with 10^4^ FFU WNV (NY99–35262 strain)	(43)
	**↑**	WNV	mRNA level (8 and 10 dpi)	C57BL/6J mice, 5–9 w old, male and female, were sc inoculated (footpad injection) with 10^2^ PFU of WNV (lineage I WNV strain (3000.0259))	(84)
	**↑**	WNV	Protein level (7 dpi)	C57BL/6 mice, 8–16 w old, male and female, were intranasally inoculated with 6 x 10^4^ PFU of WNV (Sarafend)	(101)
	**↑**	SFV	mRNA level (3, 4, 5, 7 and 10 dpi)	C57BL/6 mice, 8–12 w old, female, were ip inoculated with 5 x 10^3^ PFU of SFV strain A7 (74) or 2 x 10^5^ PFU of SFV strain L10	(102)
CCL7	**↑**	SFV	mRNA level (3, 4, 5, 7 and 10 dpi)	C57BL/6 mice, 8–12 w old, female, were ip inoculated with 5 x 10^3^ PFU of SFV strain A7 (74) or 2 x 10^5^ PFU of SFV strain L10	(102)
	**↑**	TBEV	Protein level	CSF samples from patients with TBE	(94)
	**↑**	WNV	mRNA level (8 and 10 dpi)	C57BL/6J mice, 5–9 w old, male and female, were sc inoculated (footpad injection) with 10^2^ PFU of WNV (lineage I WNV strain (3000.0259))	(84)
CCL8	**↑**	SFV	mRNA level (3, 4, 5, 7 and 10 dpi)	C57BL/6 mice, 8–12 w old, female, were ip inoculated with 5 x 10^3^ PFU of SFV strain A7 (74) or 2 x 10^5^ PFU of SFV strain L10	(102)
**CCR5**	**↑**	JEV	mRNA level	C57BL/6 J mice, 5–6 w old, male and female, were iv inoculated with 1.7–5.0 x 10^6^ PFU of JEV (P3 strain)	(65)
	**↑**	JEV	mRNA level (8dpi)	3-4 w old BALB/c mice, male and female, were injected through tail-vein with approximately 3 x 10^5^ PFU of JEV (GP78 strain)	(46)
	**↑**	WNV	mRNA level (7 and 12 dpi)	C57BL6/J mice, 8–12 w old, female, were sc inoculated with 10^4^ FFU WNV (NY99–35262 strain)	(43)
CCR5 LIGANDS					
CCL5	**↑**	JEV	Protein level	CSF samples from patients with JE	(62)
	**↑**	JEV	mRNA level	C57BL/6 J mice, 5–6 w old, male and female, were iv inoculated with 1.7–5.0 x 10^6^ PFU of JEV (P3 strain)	(65)
	**↑**	SFV	mRNA level (3, 4, 5, 7 and 10 dpi)	C57BL/6 mice, 8–12 w old, female, were ip inoculated with 5 x 10^3^ PFU of SFV strain A7 (74) or 2 x 10^5^ PFU of SFV strain L10	(102)
	**↑**	SLEV	Protein level (5 and 7 dpi)	C57BL/6 mice, 8–12 w old, female, were ic inoculated with 1 LD_100_ of SLEV (SLEV BeH 355964 strain)	(76)
	**↑**	TBEV	Protein level	CSF samples of patients with TBE	(57)
	**↑**	USUV	Protein level (6 dpi)	C57BL6/J mice, 8–12 w old, male and female, were ic inoculated with 10^4^ PFU USUV (MK 230892 strain)	(52)
	**↑**	WNV	mRNA level (7 and 12 dpi)	C57BL6/J mice, 8–12 w old, female, were sc inoculated with 10^4^ FFU WNV (NY99–35262 strain)	(43)
	**↑**	WNV	mRNA level (8 and 10 dpi)	C57BL/6J mice, 5–9 w old, male and female, were sc inoculated (footpad injection) with 10^2^ PFU of WNV (lineage I WNV strain (3000.0259))	(84)
	**↑**	ZIKV	Protein level	SWISS mice, 8–12 w old, were intra-placentally inoculated with 10^5^ PFU/mL ZIKV (PHL_2012; THA_2014; BRA_2015) Brain homogenates of E14.5 and E18.5	(38)
CCL3	**↑**	JEV	mRNA level	C57BL/6 J mice, 5–6 w old, male and female, were iv inoculated with 1.7–5.0 x 10^6^ PFU of JEV (P3 strain)	(65)
	**↑**	SFV	mRNA level (3, 4, 5, 7 and 10 dpi)	C57BL/6 mice, 8–12 w old, female, were ip inoculated with 5 x 10^3^ PFU of SFV strain A7 (74) or 2 x 10^5^ PFU of SFV strain L10	(102)
	**↑**	TBEV	Protein level	CSF samples of patients with TBE	(57)
	**↑**	WNV	mRNA level (7 and 12 dpi)	C57BL6/J mice, 8–12 w old, female, were sc inoculated with 10^4^ FFU WNV (NY99–35262 strain)	(43)
	**↑**	ZIKV	Protein level	SWISS mice, 8–12 w old, were intra-placentally inoculated with 10^5^ PFU/mL ZIKV (PHL_2012; THA_2014) Brain homogenates of E14.5 and E18.5	(38)
CCL4	**↑**	SFV	mRNA level (3, 4, 5, 7 and 10 dpi)	C57BL/6 mice, 8–12 w old, female, were ip inoculated with 5 x 10^3^ PFU of SFV strain A7 (74) or 2 x 10^5^ PFU of SFV strain L10	(102)
	**↑**	TBEV	Protein level	CSF samples of patients with TBE	(57)
	**↑**	WNV	mRNA level (7 and 12 dpi)	C57BL6/J mice, 8–12 w old, female, were sc inoculated with 10^4^ FFU WNV (NY99–35262 strain)	(43)
CXCR2 LIGANDS					
CXCL8	**↑**	JEV	Protein level	CSF samples of patients with JE	(79)
	**↑**	TBEV	Protein level	CSF samples of patients with TBE	(103)
CXCL1	**↑**	MVEV	mRNA level (6, 7 and 8 dpi)	Swiss mice, 21 days old, male and female, were ip inoculated with 10^4^ PFU MVEV (BH3479 strain)	(78)
	=	SFV	mRNA level (3, 4, 5, 7 and 10 dpi)	C57BL/6 mice, 8–12 w old, female, were ip inoculated with 5 x 10^3^ PFU of SFV strain A7 (74) or 2 x 10^5^ PFU of SFV strain L10	(102)
	**↑**	SLEV	Protein level (5 and 7 dpi)	C57BL/6 mice, 8–12 w old, female, were ic inoculated with 1 LD_100_ of SLEV (SLEV BeH 355964 strain)	(76)
	**↑**	TBEV	Protein level	CSF samples of patients with TBE	(103)
	**↑**	USUV	Protein level (6 dpi)	C57BL6/J mice, 8–12 w old, male and female, were ic inoculated with 10^4^ PFU USUV (MK 230892 strain)	(52)
CXCL2	**↑**	TBEV	Protein level	CSF samples of patients with TBE	(103)
	**↑**	WNV	mRNA level (7 and 12 dpi)	C57BL6/J mice, 8–12 w old, female, were sc inoculated with 10^4^ FFU WNV (NY99–35262 strain)	(43)
**CXCR3**	**↑**	JEV	mRNA level (8dpi)	Three to four weeks old BALB/c mice male and female, were injected through tail-vein with approximately 3 x 10^5^ PFU of JE virus (GP78 strain)	(46)
	**↑**	WNV	mRNA level (7 and 12 dpi)	C57BL6/J mice, 8–12 w old, female, were sc inoculated with 10^4^ FFU WNV (NY99–35262 strain)	(43)
CXCR3 LIGANDS					
CXCL9	**↑**	SFV	mRNA level (3, 4, 5, 7 and 10 dpi)	C57BL/6 mice, 8–12 w old, female, were ip inoculated with h 5 x 10^3^ PFU of SFV strain A7 (74) or 2 x 10^5^ PFU of SFV strain L10	(102)
	**↑**	WNV	mRNA level (8 and 10 dpi)	C57BL/6J mice, 5–9 w old, male and female, were sc inoculated (footpad injection) with 10^2^ PFU of WNV (lineage I WNV strain (3000.0259))	(84)
CXCL10	**↑**	JEV	Protein level	CSF samples from patients with JE	(62)
	**↑**	SFV	mRNA level (3, 4, 5, 7 and 10 dpi)	C57BL/6 mice, 8–12 w old, female, were ip inoculated with 5 x 10^3^ PFU of SFV strain A7 (74) or 2 x 10^5^ PFU of SFV strain L10	(102)
	**↑**	USUV	Protein level (6 dpi)	C57BL6/J mice, 8–12 w old, male and female, were ic inoculated with 10^4^ PFU USUV (MK 230892 strain)	(52)
	**↑**	WNV	mRNA level (7 and 12 dpi)	C57BL6/J mice, 8–12 w old, female, were sc inoculated with 10^4^ FFU WNV (NY99–35262 strain)	(43)
	**↑**	WNV	mRNA level (8 dpi)	C57BL/6J mice, 5–9 w old, male and female, were sc inoculated (footpad injection) with 10^2^ PFU of WNV (lineage 1 WNV strain (3000.0259))	(84)
	**↑**	ZIKV	Protein level	SWISS mice, 8–12 w old, were intra-placentally inoculated with 10^5^ PFU/mL ZIKV (PHL_2012; THA_2014) Brain homogenates of E14.5 and E18.5	(38)
**CXCR4**	**↑**	WNV	mRNA levels (8 dpi)	C57BL/6J mice, 5 w old, male and female, were sc inoculated (footpad injection) with 10 PFU of WNV lineage 1 WNV strain (3000.0259))	(93)
CXCR4 LIGANDS					
CXCL12	=	SFV	mRNA level (3, 4, 5, 7 and 10 dpi)	C57BL/6 mice, 8–12 w old, female, were ip inoculated with h 5 x 10^3^ PFU of SFV strain A7 (74) or 2 x 10^5^ PFU of SFV strain L10	(102)
	**↑**	TBEV	Protein level	CSF concentrations TBE patients	(94)
	↓	WNV	mRNA levels (at 8 dpi)	C57BL/6J mice, 5 w old, male and female, were sc inoculated (footpad injection) with 10 PFU of WNV lineage 1 WNV strain (3000.0259))	(93)
	**↑**	ZIKV	Protein level	CSF samples from Indian origin rhesus macaques that were sc inoculated with 10^4^ PFU of ZIKV (ZIKV Rio U-1/2016 (KU926309))	(90)
**CX3CR1**	**↑**	WNV	mRNA level (7 and 12 dpi)	C57BL6/J mice, 8–12 w old, female, were sc inoculated with 10^4^ FFU WNV (NY99–35262 strain)	(43)
CX3CR1 LIGANDS					
CX3CL1	=	SFV	mRNA level (3, 4, 5, 7 and 10 dpi)	C57BL/6 mice, 8–12 w old, female, were ip inoculated with 5 x 10^3^ PFU of SFV strain A7 (74) or 2 x 10^5^ PFU of SFV strain L10	(102)

CSF: cerebrospinal fluid, dpi: days post infection, FFU: focus-forming units, ic: intracranial, ip: intraperitoneal, JE: Japanese encephalitis, JEV: Japanese encephalitis virus, PFU: plaque-forming units, sc: subcutaneous, SFV: Semliki Forest virus, SLEV: Saint Louis encephalitis virus, TBE: Tick-borne encephalitis, TBEV: Tick-borne encephalitis virus, WNV: West Nile virus, ZIKV: Zika virus, USUV: Usutu virus

Bold text are chemokine receptors.

Notably, the chemokine system is highly redundant, meaning that multiple chemokines signal through the same receptor and a single chemokine can activate different receptors. This redundancy ensures robust leukocyte recruitment but also complicates therapeutic targeting. On top of that, closely related neurotropic flavivirus can induce distinct patterns of chemokine expression and leukocyte infiltration (38, 39). In the following sections, we will discuss findings regarding chemokine receptors and their ligands during flaviviral encephalitis.

### 3.1 CCR2

CCR2 is a chemokine receptor that plays a crucial role in regulating monocyte egress from the bone marrow and their trafficking to sites of inflammation. CCR2 is highly expressed on inflammatory monocytes and is found on subsets of activated T cells, dendritic cells (DC) and natural killer (NK) cells (40, 41). The primary ligand of CCR2 is CCL2, which is significantly increased during flavivirus infections (42, 43). During JEV infection, elevated levels of CCL2 were observed in distinct brain regions including the cortex, striatum, thalamus, hippocampus, sub-ventricular zone and midbrain in both rat and mouse models. Additionally, JEV-infected mice showed increased CCR2 expression compared to mock controls (44–47). Monocytes and macrophages are the main sources of CCL2, but many cell types produce this chemokine, including endothelial cells, fibroblasts, epithelial cells, smooth muscle cells, mesangial cells, neurons, astrocytes and microglia (48).

CCR2^-/-^ mice are often used to study functions of monocytes *in vivo*. For instance, CCR2 deficiency in WNV-infected mice increased mortality and viral load in the brain due to the lack of accumulating monocytes in the periphery during the early stages of WNV infection (49). Although some studies suggest that CCR2 is dispensable for monocyte trafficking from the blood to the brain, other research indicates that CCR2-expressing monocytes infiltrate the CNS more efficiently than CCR2-deficient monocytes (50, 51). Nevertheless, CCR2 deficiency alone was sufficient to completely block inflammatory monocyte recruitment to the brain in mice intracranially infected with USUV (52).

A recent study suggested that CCR2 is not required for the recruitment of T cells and viral control in acute WNV infection. However, the receptor does play a role in the recovery phase by regulating interferon-γ (IFN-γ) production by CD8^+^ T cells (53). Nevertheless, in the context of JEV infection, numerous CD8^+^ T cells expressing CCR2 and CCR5 infiltrate into the CNS.

In the context of JEV infection, CCR2 plays an interesting role: CCR2 ablation increases resistance, while CCL2 ablation increases susceptibility. This indicates that blocking CCR2, but not CCL2, could be a potential strategy for prophylaxis and therapy against JEV infection. However, the recruitment of inflammatory monocytes is not solely dependent on the CCL2-CCR2 axis, as other CCR2 ligands, such as CCL7, CCL8, CCL12 and CCL13, may also contribute (40–42).

In addition to its role in cell migration, CCR2 potentially contributes to BBB disruption. Endothelial CCR2 signaling alters TJ proteins, resulting in increased endothelial permeability *in vitro* and *in vivo*. Moreover CCR2-driven BBB permeability can be augmented indirectly through the recruitment of leukocytes (54). Notably, in a mouse model of USUV encephalitis, CCR2 deficient mice were protected against BBB disruption (52). These studies indicate that the role of CCR2 and CCL2 in the progression of viral encephalitis appears to be complex and context-dependent.

### 3.2 CCR5

CCR5 has been extensively studied for its role as the co-receptor for human immunodeficiency virus (HIV), but it was also the first chemokine receptor recognized to play an important role in flavivirus encephalitis (55). In contrast to HIV, the loss-of-function CCR5-Δ32 genetic variant was identified as a risk factor in WNV and TBEV infections. This led to the hypothesis that CCR5 deficiency is a contributing factor to symptomatic flavivirus infection due to the lack of its function in early peripheral clearance and driving lymphocyte migration into the CNS, resulting in severe disease (43, 56–59). Besides driving lymphocyte migration, CCR5 is involved in the CNS recruitment of monocytes, NK cells and the homing of CD4^+^ regulatory T cells (43, 60, 61).

CCL5 is the primary ligand of CCR5, although CCL3 and CCL4 are also recognized as cognate ligands. Elevated levels of CCL3 and CCL5 were observed in embryonic brains following intra-placental infection with various ZIKV strains (38). Furthermore, elevated levels of CCL5 in CSF were also detected in patients with JEV infection, with higher levels observed in non-surviving patients (62).

In addition, increased CCL5 production was observed in patients with mild TBEV symptoms, even without intrathecal inflammation, whereas patients with TBEV-induced meningoencephalitis showed elevated CCL5 levels in the CSF, which correlated with pleocytosis (57). Moreover, recent findings have identified higher recruitment and expansion of CD4^+^ and CD8^+^ T cells and B cells to be associated with more severe disease and neurological damage. CD4^+^ T cells are linked to encephalopathy, myelitis and cerebellar syndrome, while CD8^+^ T cells and B cells are associated with myelitis and encephalopathy (64). During JEV infection, CCR5 expression was dramatically increased in infiltrated CD8^+^ T cells, along with increased production of tumor necrosis factor-α (TNF-α), IFN-γ and granzyme B, indicating their activation and potential role in cell killing and viral clearance (65). However, CD8^+^ T cells can also contribute to immunopathology by mounting excessive cytotoxic responses that lead to tissue damage, as suggested by improved survival in TBEV-infected SCID mice, which lack functional T cells (66). Likewise, excessive production of proinflammatory cytokines by CD4^+^ T cells might increase disease severity in the context of high viral titers. Nevertheless, CD4^+^ T cells are critical for survival, as their absence in mice resulted in persistent CNS infection and uniform mortality following WNV infection (67).

### 3.3 CCR7

Leukocytes infiltrating into the CNS during viral encephalitis utilize a distinct combination of chemotactic and homing receptors to achieve selective trafficking toward the site of inflammation. While CCR2 and CCR5 serve as chemotactic receptors, CCR7 is generally described as a homing chemokine receptor. CCR7 is expressed on DCs, monocytes, neutrophils, T and B cells. During homeostasis, CCR7 regulates the migration of T cells into secondary lymphoid organs, where its cognate ligands CCL19 and CCL21 are abundantly present. Furthermore, DCs upregulate CCR7 following activation, which allows their efficient entry into terminal lymphatics expressing CCL21, promoting efficient interaction between DC and T cells (68). During viral infections, effector immune cells release IFN-γ, which reduces CCL19 and CCL21 levels in the T cell zones of secondary lymphoid organs (69). Combined with lower CCR7 expression on mature/effector T cells, this leads to the release of these cells into circulation, allowing them to migrate towards the site of inflammation (68).

Importantly, CCR7 plays a crucial role in limiting fatal WNV encephalitis in mice, by controlling leukocyte infiltration into the CNS. Loss of CCR7 led to persistent leukocytosis and increased leukocyte recruitment to the brain. Despite an excess of virus-specific T cells, CCR7-deficient mice exhibited higher viral loads and increased mortality. In addition, increased trafficking of infected myeloid cells into the brain of CCR7-deficient mice resulted in higher titers of WNV in the CNS (70, 71). Notably, CCR7 has been implicated in viral transmigration across the BBB in the context of the Trojan horse entry mechanism. *In vitro* models that mimic the transmigration of monocytes through BBB showed an upregulation of CCR7 on ZIKV-, WNV- and USUV-infected monocytes (39, 72).

### 3.4 CXCR2

Neutrophils are a critical component of the innate immune response, however, they play an underrecognized role in the pathogenesis of neurotropic flavivirus infections (73). CXCL8, CXCL1, CXCL2 and CXCL5 drive CXCR2-dependent neutrophil migration from the bone marrow into the blood. Importantly, the CXCL1–CXCR2 axis is also essential for neutrophil transendothelial migration into the brain (74). Macrophages rapidly upregulate CXCL1 and CXCL2 expression, while neurons and astrocytes also produce CXCL1 *in vivo* following HSV-1 infection which is the most common cause of viral encephalitis, although not a flavivirus (75). In mouse models of neurotropic flavivirus infections such as SLEV and MVE, neutrophil infiltration into the CNS was preceded by increased expression of CXCL1 within the CNS (76–78). A similar pattern was observed in patients, with higher CSF levels of CXCL8 associated with more severe outcomes of JE (79). Beyond their traditional immune functions, CXCR2^+^ neutrophils can act as viral reservoirs, supporting WNV replication and facilitating viral dissemination early during infection (74, 75).

In addition to its chemotactic function, CXCR2 also mediates the activation of the CNS endothelium. Mice deficient in either CXCL1 or CXCR2 show reduced leukocyte and endothelial interactions and lower expression of CAMs such as P-selectin, VCAM-1, and ICAM-1 in the brain following lipopolysaccharide (LPS) injection. Furthermore, *in vitro* stimulation of brain microvascular endothelial cells with CXCL1 increases BBB permeability and promotes monocyte transendothelial migration (80). This was also observed upon JEV infection, which led to activation of brain microvascular endothelial cells and the release of CXCL1 (63).

### 3.5 CXCR3

CXCR3 is preferentially expressed on activated T cells, particularly the T helper 1 (Th1) subset, NK cells and B cells. CXCR3 is activated by three highly related chemokines: CXCL9, CXCL10 and CXCL11, which are induced by CNS challenges such as neurotropic infections. Within the CNS, microglia and astrocytes are the main source of CXCL9 and CXCL10 (81, 82). The CXCL10-CXCR3 axis is particularly important in the context of WNV infection in the CNS, as reviewed by Benzarti et al. (83). In a mouse model of WNV encephalitis, neurons were identified as an additional source of CXCL10, inducing recruitment of CD8^+^ T cells (84). Similarly, CXCL10 facilitates T cell entry into the CNS parenchyma during TBEV infection. Patients with meningitis, encephalitis or meningoencephalitis caused by TBEV showed increased concentrations of CXCL10 and CXCL11 in the CSF and elevated CXCL10 levels in blood, indicating their potential role as biomarkers of inflammatory processes in the CNS. Moreover, the regression of CXCL10 in the CSF was proposed as an indicator of patient recovery during TBEV infection (85, 86).

Another ligand that is able to activate CXCR3 is CXCL4, also known as platelet factor 4 due to its release in high concentrations upon platelet activation. Elevated levels of CXCL4 have been observed during JEV infection, where it interacts with CXCR3 or CXCR3B, a splice variant of CXCR3, on vascular endothelial cells, epithelial cells, fibroblasts and leukocytes (87). Recent research highlighted the therapeutic potential of targeting the CXCR3 pathway. Blocking CXCR3 with the antagonist AMG487 in JEV-infected mice elevated the expression of type 1 IFNs, hereby reducing viral replication and increasing secretion of proinflammatory cytokines and leukocyte infiltration (88). Similar mechanisms were seen when infecting CXCL4-deficient mice with JEV. The suppression of the inflammatory response in these mice was associated with reduced percentages of monocytes, macrophages and T cells, along with decreased levels of proinflammatory cytokines in the brain compared to wild type (WT) mice (89).

### 3.6 CXCR4

CXCR4 is a chemokine receptor that is widely expressed on both immune- and non-immune cells, including hematopoietic cells, endothelial cells, neurons and stem cells. CXCR4 and its exclusive ligand CXCL12 are involved in numerous physiological and pathological conditions, including neurogenesis, immune responses and vascularization (90, 91). The constitutive expression of CXCL12 along the basolateral surfaces of CNS endothelial cells and within the brain microvasculature maintains an immune-privileged environment. This expression retains T cells, which commonly express CXCR4, in the perivascular space after crossing the BBB, thereby hindering CD8^+^ T cells from effectively clearing WNV within the CNS parenchyma (92). The antagonism of CXCR4 improved survival from lethal infection with WNV through enhanced intraparenchymal migration of WNV-specific CD8^+^ T cells, leading to reduced viral load and decreased immunopathology at this site (93). CXCR4 is expressed on inflammatory monocytes, but the CXCL12-CXCR4 axis is not involved in monocyte recruitment in TBE. Nevertheless, CXCL12 was synthesized intrathecally and detected in the periphery before the onset of pleocytosis, suggesting that this chemokine may contribute to CNS inflammation in its earliest stages (94). However, CXCL12 expression in the brain during WNV encephalitis depended on the mouse model used (83). Nevertheless, in adult Indian rhesus macaques, subcutaneous ZIKV infection caused increased CXCL12 expression in the CNS, which persisted long after the initial infection was cleared. In addition, CXCL12 plays a crucial role in regulating lymphocyte trafficking across the BBB into the CNS and in promoting the repair of damaged neural tissue, including remyelination (90).

### 3.7 CX3CR1

The chemokine CX3CL1 (fractalkine) is constitutively expressed by healthy neurons and endothelial cells (95, 96). CX3CR1 is expressed on resident brain cells, including microglia and astrocytes, as well as several leukocytes, such as DCs, monocytes, NK cells and T cells. The function of CX3CL1 varies depending on its form: soluble CX3CL1 acts as a chemoattractant, whereas membrane-anchored CX3CL1 functions as a CAM that facilitates the interaction of microglia and infiltrating leukocytes during inflammation (97). Binding of CX3CL1 to CX3CR1 on microglia provides potent inhibitory signals that help maintain their quiescent state, thus serving as a key mechanism by which neurons regulate innate immunity in the CNS (98). In addition, the soluble CX3CL1-CX3CR1 axis induces survival pathways in monocytes (99).

Studies investigating the role of CX3CR1 in the pathogenesis of neurotropic flavivirus-induced encephalitis are limited. Nevertheless, in WNV-infected mice, CX3CR1 and its ligand were upregulated in the brain by 7 days post infection (43). However, in later stages of disease, CX3CR1 expression on microglia was downregulated and these cells underwent apoptosis (100). Additionally, CX3CR1^+^ DC activation following JEV infection triggers their migration to the draining lymph nodes, promoting robust antiviral NK cell activity and virus-specific T cell responses (98).

## 4 Other chemoattractants

In addition to chemokines, other chemoattractants contribute to leukocyte recruitment during viral encephalitis. These include bioactive lipids (e.g., leukotriene B4 (LTB4)), complement anaphylatoxins (e.g., C3a and C5a) and find-me signals (e.g., nucleotides released by dying cells). In contrast to chemokines, these molecules do not depend on GAG binding and bind to specific receptors in a non-redundant manner.

### 4.1 Bioactive lipids

Leukotrienes are lipid mediators derived of the arachidonic acid metabolism via the 5-lipoxygenase pathway. LTB4 is a potent chemoattractant that activates leukocytes by binding to its high affinity receptor leukotriene B4 receptor 1 (BLT1). LTB4 synthesis is induced in response to other cytokines, such as IL-1 and TNF-α, bacterial products and other chemoattractants (platelet-activating factor (PAF) and C5a). Notably, during viral infection, there is evidence that leukotriene production is upregulated (104). In a mouse model of JEV infection, treatment with fenofibrate, which is a peroxisome proliferator-activated receptor-α (PPAR-α) agonist, reduced the levels of LTB4 in the brain. Fenofibrata acts by inducing the expression of cytochrome P450 4F (Cyp4f) enzymes, which catabolize LTB4 through hydroxylation (105).

Another notable lipid mediator is PAF, a potent proinflammatory phospholipid that is biosynthesized from phosphatidylcholine. The production of PAF and the subsequent release from platelets and leukocytes (neutrophils, macrophages and B cells) is induced by several cytokines such as IL-1, IL-6, IL-12 and TNF-α. PAF has pleiotropic effects after binding to its PAF receptor (PAFR), activating several proinflammatory signaling pathways including NF-κB and protein kinase C (106, 107).

PAF and LTB4 are often concomitantly produced and have been described in the recruitment of neutrophils upon stimulation with diverse toll like receptor (TLR) ligands (108). Although the production of PAF has not been studied in neurotropic flaviviruses yet, a previous study showed that macrophages from individuals with prior DENV infection increased PAF production compared to those from non-infected individuals (109). Therefore, excessive activation of PAFR was hypothesized to worsen symptoms of dengue hemorrhagic fever and dengue shock syndrome. Indeed, in PAFR-deficient mice inoculated with DENV, there was a decrease in thrombocytopenia, hyperpermeability and systemic cytokine levels, as well as the delay of mortality compared to infected WT mice (110).

### 4.2 Complement anaphylatoxins

The complement system is a large network of soluble and membrane-associated proteins that play an essential role in the innate immune response. Complement fragments C3a and C5a mediate leukocyte recruitment through C3aR and C5aR, respectively. C3aR is expressed on monocytes, activated T cells, neutrophils, basophils, eosinophils and mast cells. In the CNS, C3aR is constitutively expressed on neurons and during inflammation on astrocytes and microglia as well. C3aR-mediated chemotaxis was shown for monocytes, eosinophils and mast cells, but not neutrophils (111). C5aR expression is much more abundant than C3aR, and was shown on neutrophils, monocytes, T cells, B cells, DCs and mast cells. Evidence for C5aR-induced chemotaxis is available for neutrophils, monocytes, DCs, T cells and B cells (111). C5aR signaling in neutrophils also mediates cell recruitment indirectly via the release of LTB4 and upregulation of CAMs (112).

Even though it is clear that complement is activated upon flavivirus infection, its function during disease remains unclear. CSF proteome analysis of JEV-infected patients showed elevated levels of C3 (113). Mice deficient in C1q, C3, C4, factor B or factor D that were infected subcutaneously with WNV all showed decreased survival and increased viral burden in the brain and spinal cord (114, 115). However, C5aR-deficient mice shared similar susceptibility to WNV infection as WT mice. Importantly, this study showed that complement activation is required for T cell migration to the brain after peripheral WNV infection. Aberrant recruitment of both CD4^+^ and CD8^+^ T cells in C4 and factor B deficient mice, but not in C1q deficient mice suggests that the lectin and alternative pathways of complement activation are required (115).

Interestingly, viruses have developed specific strategies to antagonize complement activation. For instance, WNV, DENV and YF non-structural protein 1 (NS1) inhibits classical and lectin pathway activation through direct binding of C4 and promoting its cleavage to C4b (116). Moreover, WNV NS1 was shown to further inhibit the alternative pathway of complement activation by binding regulatory factor H (117).

Adverse effects of complement proteins in the brain have also been described following flavivirus neuroinvasion. Murine C3 levels in the brain are significantly upregulated following intracranial ZIKV infection. Neutralization with C3-specific antibodies rescued ZIKV-induced synapse pathology in the hippocampus and memory impairment (118). Likewise, C3 expression is highly increased following intracranial WNV infection and C3 or C3aR deficient mice are protected from synaptic loss in the hippocampus (119).

### 4.3 Find-me signals

Neurotropic flavivirus infections lead to non-lytic cell death by apoptosis and autophagy of neurons and, in some cases, glial cells, contributing to neurological dysfunction (120, 121). Clearance of apoptotic cells is mediated by find-me signals, which are molecules released by dying cells that attract phagocytes. This group of molecules mainly comprises the lipids lysophosphatidylcholine, sphingosine 1-phosphate, the chemokine CX3CL1 (vide supra), and the nucleotides ATP and UTP (122, 123). In-depth understanding of the mechanisms by which these mediators function is still required. Nevertheless, recent studies have reported an increase in LPC levels in the plasma of TBE patients as well as WNV-infected mice (124, 125).

Necroptosis is another type of cell death and was recently found to be a cause for neuronal loss in JEV-infected brains (126, 127). Moreover, WNV induces expression of necroptosis and pyroptosis cell death markers in the brain of infected mice. These programmed lytic forms of cell death release damage-associated molecular patterns (DAMPs) which are highly inflammatory. For instance, high mobility group **Box 1** (HMGB1) protein is an abundant nuclear component released upon membrane permeabilization and secreted by activated monocytes, macrophages, DCs, endothelial cells and platelets (128). Once released, HMGB1 binds to receptors for advanced glycation end products (RAGE), TLR2 and TLR4, which are expressed by many cell types including neurons, endothelial cells and leukocytes (129, 130). HMGB1 induces multiple signaling pathways, leading to cell migration directly or inducing the transcription of cytokines and chemokines, stimulating immune cells in an autocrine and paracrine feedback loop (131, 132). Moreover, HMGB1 promotes CXCR4-dependent cell migration by upregulating CXCL12 via NF-κB and forming HMGB1-CXCL12 heterocomplexes, which protects CXCL12 degradation and induces CXCR4 signaling beyond CXCL12 alone (133).

HMGB1 also plays a role in leukocyte adhesion. ZIKV-infected monocytes were shown to release HMGB1 and upregulate RAGE expression in both monocytes and human brain microvascular endothelial cells (hBMECs) in coculture. This suggests that monocyte migration across the BBB is RAGE-dependent, with HMGB1 contributing to TJ disruption (72).

Another chemokine-like molecule is macrophage migration-inhibitory factor (MIF), which is produced by various cell types including immune cells, neurons, fibroblasts and endothelial cells. MIF receptors include CD74, CXCR2, CXCR4 and CXCR7, which form receptor complexes in order to establish MIF signaling and subsequent leukocyte migration (134, 135). Even though chemoattractant function of MIF in flavivirus encephalitis has not been studied, its plasma and CSF levels were increased in patients suffering from TBEV and WNV infection (136, 137). MIF mRNA levels are upregulated in the brain of JEV-infected mice and hBMECs stimulated with JEV NS1/NS1’ (138, 139). MIF is considered pathogenic in flavivirus infections as MIF-deficient mice were protected in WNV infection and showed lower viral loads and inflammatory responses. The detrimental role of MIF is attributed to disruption of the BBB favoring viral neuroinvasion and induced expression of chemokines and CAMs on endothelial cells and monocytes (136). Importantly, another study reported an association between high-expression *MIF* alleles and severe encephalitis in patients with WNV encephalitis (140).

Potent chemoattractant formyl peptides such as N-Formylmethionyl-leucyl-phenylalanine (fMLF) originate from both bacteria and endogenous mitochondria and have been described in neurodegenerative diseases (141). However, there are no reports describing fMLF or other formyl peptides in the context of viral encephalitis and their potential contribution to leukocyte recruitment to the CNS.

## 5 Cellular adhesion molecules

**Figure 3:**
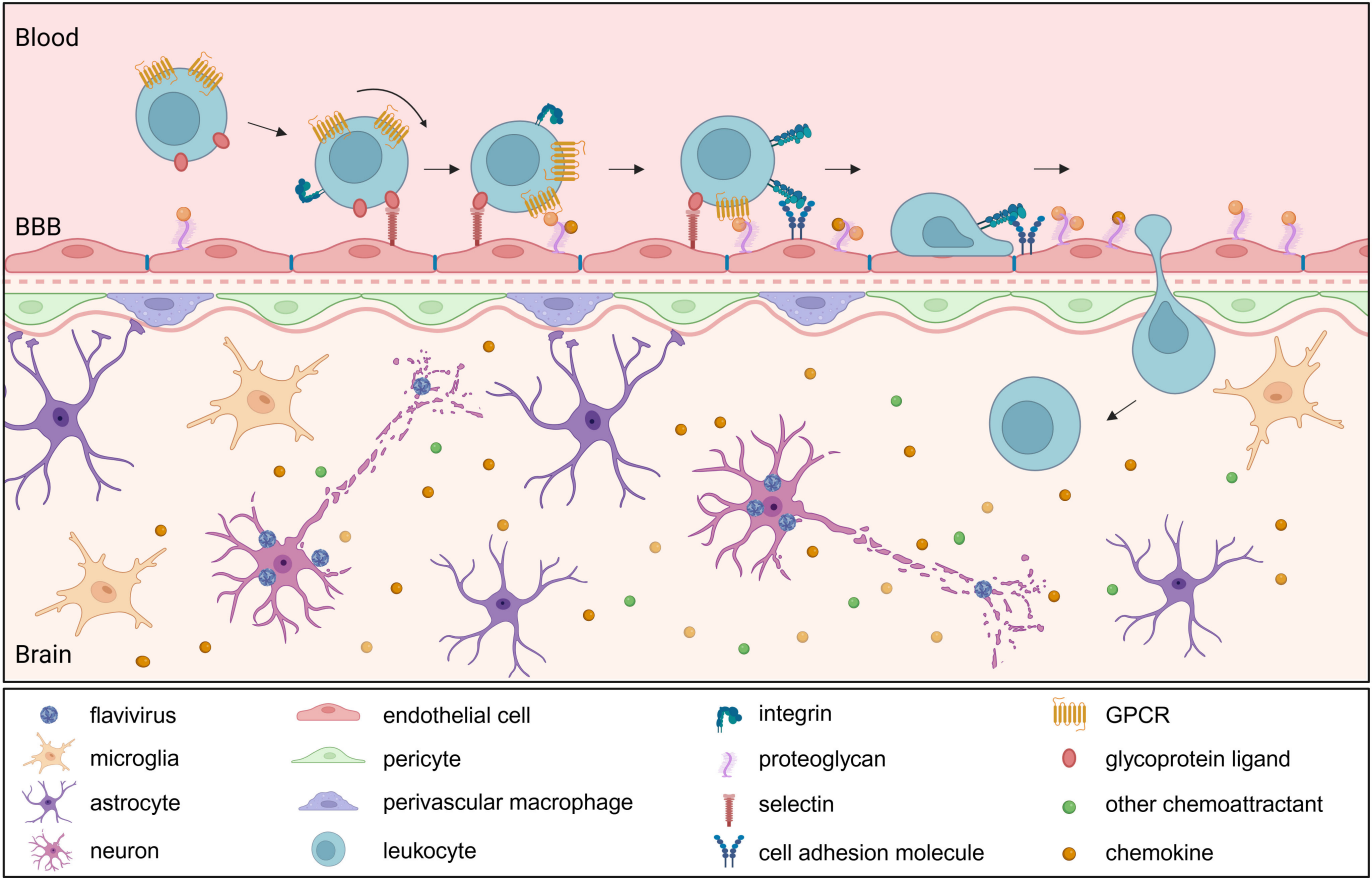
Leukocyte infiltration into the brain following neurotropic flavivirus infection. Upon neurotropic flavivirus infection, resident brain cells release chemokines (e.g., CCL2, CCL5), creating a gradient that drives leukocyte recruitment through binding to their cognate receptors (e.g., CCR2, CCR5). Activated endothelial cells upregulate adhesion molecules and present bound chemoattractants, initiating leukocyte tethering and rolling via selectin-ligand interactions. Chemoattractant receptor activation triggers integrin upregulation and activation, facilitating firm adhesion and subsequent transmigration. Once within the brain parenchyma, infiltrating immune cells amplify the inflammatory response. BBB, blood brain barrier; GPCR, G protein-coupled receptor.

Leukocyte recruitment into the CNS is crucial for the antiviral defense during viral encephalitis. In addition to chemoattractants, this process requires CAMs. These transmembrane glycoproteins mediate adhesion between leukocytes and the endothelium of the vasculature, facilitating leukocyte tethering, rolling, firm adhesion and transmigration across the BBB into the CNS. CAMs are generally divided in five subgroups: selectins, integrins, cadherins, members of the immunoglobulin superfamily and others such as mucins (142, 143). The initial attachment and rolling are mediated by binding of endothelial E- and P-selectins to their ligands on leukocytes, including L-selectin, PSGL-1, CD44, CD43 and E-selectin ligand-(ESL)1 (144). These transient interactions slow down leukocytes as they roll along the vessel wall but remain reversible unless followed by signals from chemokines presented on endothelial proteoglycans. Chemokine signaling triggers the inside-out activation of integrins, to switch from a bent form to an extended form that more easily binds ligands. Leukocyte integrins such as α4β1 (VLA-4) and the β2 integrins LFA-1 and Mac-1 can then bind to their respective endothelial ligands VCAM-1 and ICAM-1 (145). Once firmly adhered, leukocytes undergo flattening, likely to minimize shear forces from blood flow and crawl along the endothelium to find permissive sites for transendothelial migration. Transmigration, or diapedesis, can occur via paracellular or transcellular routes, and is supported by additional molecules such as PECAM-1, CD99 and JAMs. These interactions help guide leukocytes through the endothelial barrier and into the CNS parenchyma (**Figure 3**).

Expression of CAMs on the endothelial cell surface is upregulated by cytokines such as TNF-α and IL-1β via NF-κB activation, in response to microbial infection or tissue damage (146–148). Increased CAM expression in response to neurotropic flavivirus infections has been demonstrated in multiple *in vitro* and *in vivo* models (**Table 2**).

**Table 2:** Cell adhesion molecule expression in flaviviral encephalitis.

CAMs	Virus	Cell	Expression	Reference
C-TYPE LECTIN RECEPTORS				
DC-SIGN	USUV, WNV	hMoDCs	=	(39)
IMMUNOGLOBULIN SUPERFAMILY				
ALCAM	USUV, WNV	hBLEC	↑	(39)
		hMoDC	=	(39)
ICAM-1	JEV	hBMEC	↑	(149)
	TBEV	hPBMC	↑	(150)
	USUV	hBLEC, hMoDC	↑	(39)
	WNV	hBLEC, hBMEC, HEF, hUVEC, rBMEC	↑	(39, 63, 149, 151–153)
		hMoDC	=	(39)
	ZIKV	hBLEC	↑	(154)
ICAM-2	USUV	hBLEC, hMoDC	↑	(39)
	WNV	hBLEC, hBMEC	↑	(39, 149, 151, 152)
		hMoDC	=	(39)
ICAM-3	ZIKV	hMo	↑	(149)
PECAM	USUV	hBLEC	↑	(39)
		hMoDC	=	(39)
	WNV	hBLEC	↑	(39, 149, 151, 152)
		hBMEC, hMoDC	=	(39)
	ZIKV	hMo	↑	(149)
VCAM-1	JEV	hBMEC	↑	(149)
	USUV	hBLEC, hMoDC	↑	(39)
	WNV	hBLEC, hBMEC, hUVEC	↑	(39, 149, 151–153)
		hMoDC	=	(39)
	ZIKV	hBLEC	↑	(154)
INTEGRINS				
ITGA/B	USUV, WNV	hMoDCs	=	(39)
	ZIKV	hMo	↑	(149)
ITGAM	TBEV	hPBMC	↑	(150)
LFA-1	JEV	mPBMC	↑	(149)
	WNV	hMo	=	(155)
	ZIKV	hMo	↑	(149)
VLA-4	JEV	mPBMC	↑	(149)
	WNV	hMo	=	(155)
SELECTINS				
E-selectin	USUV	hBLEC, hMoDC	↑	(39)
	WNV	hBLEC, hBMEC, hUVEC	↑	(39, 149, 151–153)
		hMoDC	=	(39)
L-selectin	WNV	hBMEC	=	(149, 151, 152),
P-selectin	USUV	hBLEC, hMoDC	↑	(39)
	WNV	hBLEC	↑	(39)
		hBMEC, hUVEC, hMoDC	=	(39, 149, 151–153)

ALCAM, activated leukocyte cell adhesion molecule; DC-SIGN, dendritic cell-specific intracellular adhesion molecule-3-grabbing non-integrin; E-selectin, endothelial-selectin; HEF, human embryonic fibroblast; hBLEC, human brain like endothelial cell; hBMEC, human brain microvascular endothelial cell; hMo, human monocyte; hMoDC, human monocyte-derived dendritic cells; hPBMC, human peripheral blood mononuclear cell; hUVEC, human umbilical vein endothelial cell; ICAM, intercellular adhesion molecule; ITGA, integrin α subunit; ITGAM, integrin αMβ2; ITGB, integrin β subunit; JEV, Japanese encephalitis virus; L-selectin, leukocyte-selectin; LFA, lymphocyte function-associated antigen/integrin αLβ2; m, mouse; P-selectin, platelet-selectin; PECAM, platelet endothelial cell adhesion molecule; r, rat; TBEV, Tick-borne encephalitis virus; USUV, Usutu virus; VCAM, vascular cell adhesion molecule; VLA, very late antigen/α4β1 integrin; WNV, West Nile virus; ZIKV, Zika virus.

*In vitro* models have shown that human umbilical vein endothelial cells (hUVEC) infected with WNV upregulate E-selectin, ICAM-1, VCAM-1, but not P-selectin (153). Likewise, WNV-infected hBMECs showed increased mRNA levels of E-selectin, ICAM-1,VCAM-1, but not P-selectin, L-selectin and PECAM (151). However, a recent study comparing WNV and USUV infection on a hBBB model found that WNV, and to lesser extent USUV, increased mRNA levels of P-selectin, PECAM, ICAM-2 and ALCAM. Moreover, soluble E-selectin, P-selectin and ICAM-1 were detected in both apical and basolateral compartments (39).

In different mouse models of WNV, increased ICAM-1, VCAM-1 and E-selectin mRNA expression in the brain has been shown. Upregulation of these CAMs was correlated with peak WNV titers and leukocyte infiltration in the brain (151, 156). Moreover, ICAM-1 deficient mice were more resistant to lethal WNV infection, showing reduced viral load, leukocyte infiltration and neuronal damage (156). WNV is able to induce ICAM-1 expression in both a direct and an indirect cytokine-dependent manner (153). Indeed, a robust induction of CAM-inducing cytokines TNF-α and IL-1β is detected in the serum of patients with WNV fever and WNV encephalitis (157–159). The important role of ICAM-1 in leukocyte adhesion during flavivirus encephalitis is supported by research on JEV. Neutrophil and PBMC adhesion to rat brain microvascular endothelial cells (BMECs) was promoted by JEV infection. Moreover, pretreatment of the BMECs with ICAM-1 neutralizing antibodies almost completely inhibited leukocyte adhesion (63).

Moreover, endothelial-derived molecules indirectly promote leukocyte recruitment upon infection. For instance, hBMECs infected with JEV dramatically upregulate HMGB1 production, which enhances adhesion and transmigration of monocytes (149). RAGE functions as an endothelial adhesion receptor through direct interaction with the β2-integrin Mac-1 on innate leukocytes and in cooperation with ICAM-1 (160, 161). Moreover, HMGB1 activates endothelial RAGE and promotes expression of ICAM-1 and VCAM-1 (162). Additionally, HMGB1 also activates Mac-1 in a RAGE-dependent manner on neutrophils and upregulates the expression of LFA-1 and VLA-4 (149, 163). Lastly, MIF upregulates TNF-α-induced expression of endothelial E-selectin, P-selectin, ICAM-1 and VCAM-1and monocyte ICAM-1 and VCAM-1 (164, 165).

CAMs are absent in the choroid capillaries, aside from P-selectin, allowing leukocytes to enter the stroma freely. Interestingly, ICAM-1, VCAM-1 and mucosal addressin cell adhesion molecule 1 (MAdCAM-1) are expressed on the apical surface of CPEs, indicating their role in the final step of diapedesis and release in the CSF (27). Another CAM, epithelial V-like antigen is found on both apical and basal CPE surfaces and plays an important role for T cell adhesion, *in vitro* (166). Despite the recognized importance of leukocyte infiltration at the CP, the effects of flavivirus infection on CPE CAMs remains to be studied. Nevertheless, increased CAM expression by CPEs induced by TNF-α has been reported, suggesting a response in flavivirus encephalitis (26).

Flavivirus infection also increases the expression of complementary CAMs on leukocytes. However, the expression of integrins LFA-1 and VLA-4 in isolated monocytes, which are complementary to ICAM-1 and VCAM-1, respectively, did not increase upon WNV infection. Nevertheless, blocking VLA-4 reduced neutrophil, T-cell and especially monocyte counts in the brains of WNV-infected mice, alleviating encephalitic symptoms without affecting viral titers (155).

Monocytes and monocyte-derived dendritic cells (MoDCs) infected with WNV or USUV *in vitro* showed increased binding to both infected and non-infected human brain like endothelial cells (hBLECs), suggesting a direct effect of these viruses on leukocyte gene expression. The effect of USUV infection on binding ability and increased CAM expression of DCs was more pronounced compared to WNV infection (39). Likewise, ZIKV-infected monocytes showed increased efficiency in adherence, migration and transmigration assays compared to their mock-infected controls. Specifically, transmigration ability was improved by ZIKV-induced upregulation of LFA-1 (167, 168). Finally, the C-type lectin Dendritic Cell-Specific Intercellular adhesion molecule-3-Grabbing Non-integrin (DC-SIGN), which is abundantly expressed on immature DCs, has been identified as a key entry receptor for flaviviruses, interacting with glycosylation sites on the flavivirus E protein to facilitate viral entry (169).

## 6 Conclusion and future perspectives

Significant progress has been made in understanding how leukocytes infiltrate the CNS parenchyma during encephalitis (**Box 1**). However, many crucial questions persist regarding the precise mechanisms governing leukocyte recruitment into the inflamed CNS. For instance, it is still unknown which cells are producing which chemoattractants, and most lack confirmation at the protein level. The majority of the studies rely on measurements of mRNA levels of chemoattractants in whole tissue lysates. Much deeper insights would be obtained via single-cell/single-nucleus RNA sequencing and spatial transcriptomics, pinpointing the exact cellular sources for each of the molecules found during encephalitis. Importantly, RNA sequencing technologies can now be coupled to protein expression, such as CITE-seq (Cellular Indexing of Transcriptomes and Epitopes by sequencing), and techniques such as MILAN (multiple iterative labeling by antibody neodeposition) provide direct multiplexed protein identification in samples.

Box 1 **Summary of key mechanisms driving leukocyte recruitment in the CNS**

- Flaviviruses can enter the CNS via multiple routes, including the BBB, BCSFB and via infected leukocytes (Trojan horse mechanism).
- Once in the CNS, flavivirus infection triggers the release of chemoattractants.
- Chemokines guide leukocyte migration into the CNS by binding GAGs and activating specific GPCRs on leukocytes.
- Non-chemokine chemoattractants (e.g., LTB4, C5a, HMGB1) also contribute to leukocyte recruitment through distinct, non-redundant GPCR pathways.
- Different flaviviruses elicit both overlapping and virus-specific patterns of leukocyte recruitment and chemoattractant profiles.
- CAMs expressed on CNS endothelium (e.g., ICAM-1, VCAM-1, E-selectin) facilitate leukocyte adhesion, rolling and transmigration across barriers.
- Flavivirus infection modulates CAM expression on both endothelial cells and leukocytes.

It is evident that disrupting a single chemokine receptor or CAM is insufficient to completely block CNS inflammation and improve encephalitis outcomes. This is likely due to the redundant expression of multiple chemoattractants and CAMs, which can compensate for the absence of a single pathway. This highlights the importance of identifying and targeting the receptors primarily utilized by pathogenic leukocytes, while sparing those employed by resolving leukocytes. Future investigations are required to identify the most efficacious molecular targets to limit pathogenic leukocyte trafficking in encephalitis. Moreover, the distinct spatiotemporal and hierarchical contributions of chemoattractants are still elusive.

Besides attracting leukocytes, chemoattractants are also involved in cellular proliferation, which may significantly impact disease progression and recovery. Currently, this aspect of encephalitis is unknown. Multiple chemoattractants, including chemokines, also act on resident cell populations in the CNS, for instance, regulatory T cells and microglia. The impact of chemoattractant signalling locally, likely in early stages of encephalitis, is certainly worthy of investigation.
